# Electromagnetic study of a split-ring resonator metamaterial with cold-electron bolometers

**DOI:** 10.3762/bjnano.16.152

**Published:** 2025-12-05

**Authors:** Ekaterina A Matrozova, Alexander V Chiginev, Leonid S Revin, Andrey L Pankratov

**Affiliations:** 1 Nizhny Novgorod State Technical University n.a. R.E. Alekseev, MininStreet, 24, Nizhny Novgorod, 603155, Russia; 2 Institute for Physics of Microstructures of the Russian Academy of Sciences, Akademicheskaya Street, 7, Nizhny Novgorod, 603950, Russia

**Keywords:** cold-electron bolometer, metamaterial, split-ring resonator

## Abstract

We present an electromagnetic study of a metamaterial receiver based on split-ring resonators with integrated cold-electron bolometers. We suggest a modified antenna design that allows one to significantly increase the absorbed power and the bandwidth. The trade-off between the bandwidth expansion due to miniaturization and the reduction in absorption efficiency determined by the Airy spot size of the coupling lens is investigated. To solve this issue, a simultaneous miniaturization of the size of the entire structure with an increase in the number of array elements is proposed. The design with a 37-element array demonstrates an increase in power absorption by a factor of 1.4 compared to the original 19-element single-ring array, as well as an increase in operating bandwidth from 160 to 820 GHz.

## Introduction

Highly sensitive receivers with broadband antennas are of significant interest for advanced spectroscopic applications and various radioastronomy tasks [1–5]. In particular, broadband receiving systems are required for use with a Fourier-transform spectrometer based on the Martin–Paplett interferometer that is planned to be used in future missions such as BISOU (Balloon Interferometer for Spectral Observations of the Universe) [3–4] and Millimetron [2,5]. The use of cold-electron bolometers (CEBs) is particularly advantageous for such systems, enabling operation in a wide frequency range from gigahertz frequencies to X-rays [6–8] due to a normal-metal absorber. CEBs offer several advantages over other types of receivers such as transition edge sensors [9–11]. These advantages include their micrometer-scale size, which facilitates direct integration into antenna slots without the need for microwave feed lines (e.g., microstrip or coplanar lines), thus simplifying the design and preventing signal degradation at higher frequencies [12]. Furthermore, the natural electron cooling mechanism in CEBs [13–15] is highly suitable for operation with cryogenic systems such as ^3^He sorption fridges. Perhaps most critically, CEBs demonstrate exceptional hardness against cosmic rays [16], a paramount requirement for balloon and space missions.

Our group has recently designed, fabricated, and characterized a metamaterial receiver with integrated CEBs, operating in a broad frequency range [17]. In that work, each element represented a ring antenna with two embedded CEBs connected parallel in DC, whereas the antennas in the array were connected in series. In the present work, we propose and numerically investigate a new design of a CEB metamaterial receiver based on double split-ring resonators (SRRs) [18] to increase both the magnitude of the absorbed signal and the working bandwidth. We consider various geometrical modifications of this design and perform a comparative analysis.

## Design and Simulation Approach

In our previous work [17], a metamaterial comprising 19-ring antennas enabled the reception of external electromagnetic signals in the broad band from 150 to 550 GHz, as well as in the band from 900 to 1300 GHz. To further enhance signal absorption, we propose replacing simple ring antennas with SRRs [19–20,18,21]. The SRR is a well-established magnetic metamaterial element whose resonant properties are governed by its internal inductance and capacitance, allowing for a strong magnetic response and associated current loops at the designed resonance frequency.

The simulations of the metamaterial arrays were performed in the time-domain solver of CST MWS in 3D mode. The simulated receiving structure is placed on a 500 μm thick silicon substrate. A 4 mm-diameter silicon hyperhemispherical lens is placed on the rear side of the substrate to efficiently couple the incident radiation into the planar structure. The external signal is incident from the H_11_ mode of the round waveguide port located behind the Si lens, simulating a realistic excitation source. The electric field of the incident wave is directed perpendicularly to the gaps in the receiving elements.

**Figure 1:**
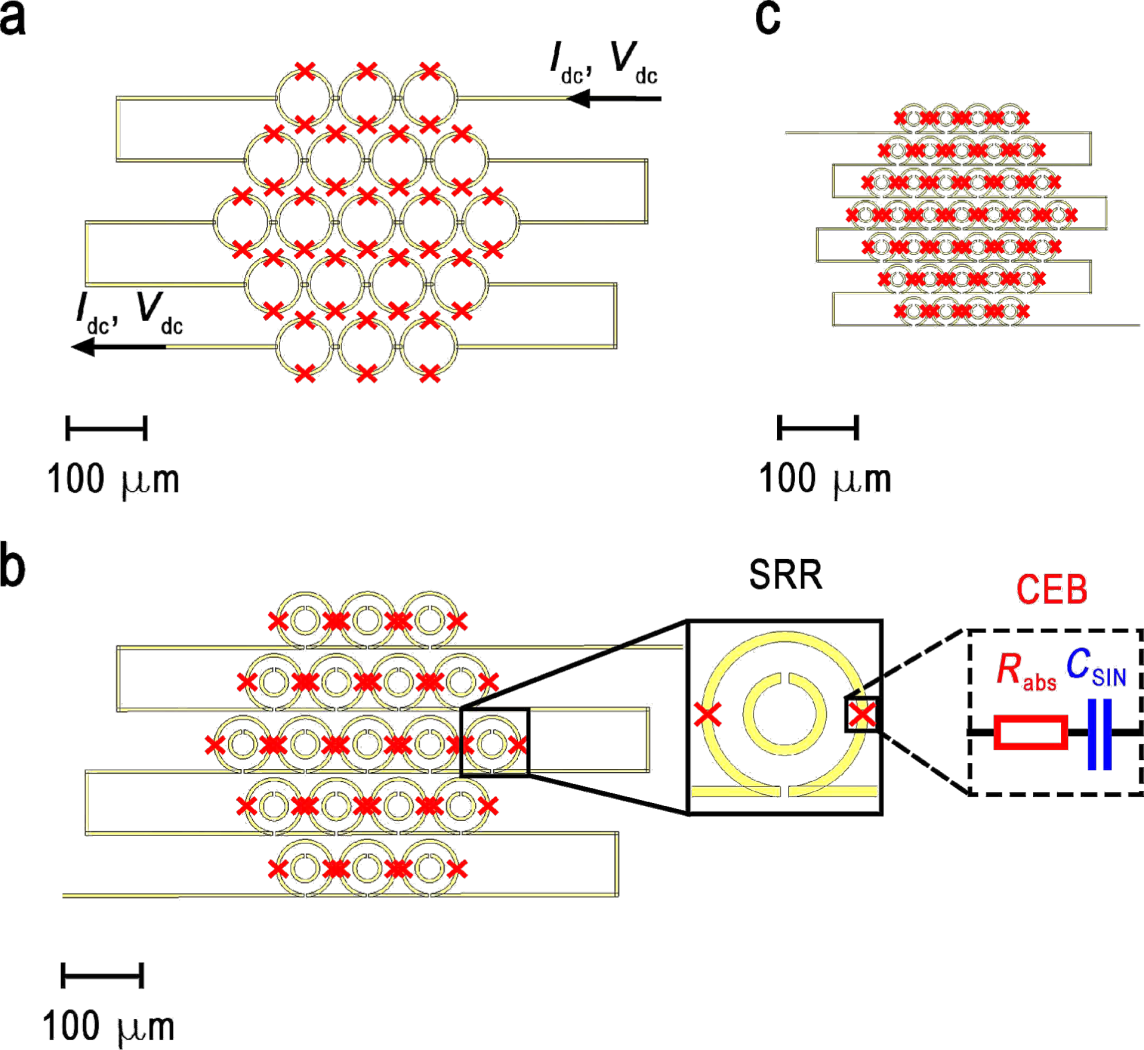
Schematic layout of the investigated metamaterial arrays. (a) 19-element array of single-ring antennas; (b) 19-element array of split-ring resonators; (c) 37-element array of miniaturized SRRs. Inset: a single unit cell with two embedded CEBs represented as an RC circuit.

The signal is received by an array of the proposed ring resonators. Two CEBs are embedded into the outer ring of each SRR element. In the simulations, each CEB is modeled as an RC circuit (see inset in Figure 1), where *R*_abs_ = 75 Ω represents the resistance of the CEB’s normal-metal absorber, and *C*_SIN_ = 20 fF is the capacitance of the two SIN junctions of the CEB connected in series. The total absorbed power is calculated as the sum of the powers absorbed in these discrete ports representing the CEBs. The power in our modeling is normalized to the power outgoing from the waveguide port, which is equal to 0.5 in arbitrary units.

## Results

The design of the previously studied metamaterial with CEBs and single-ring antennas is shown in Figure 1a. To increase the absorbed power and the working frequency band, we propose and analyze a new design based on SRRs (Figure 1b,c). The geometric parameters of the structures are as follows: A single ring has an outer ring diameter of *d*_ext_ = 80 μm and an inner ring diameter of *d*_int_ = 70 μm. The lattice constant (period) of the metamaterial array is *P* = 86 μm. The total size of the structure is 424 μm. A large-scale SRR has an outer ring with an external diameter of *d*_ext_*_,_*_1_ = 80 μm and an internal diameter of *d*_int_*_,_*_1_ = 70 μm. The inner ring has an external diameter of *d*_ext_*_,_*_2_ = 40 μm and an internal diameter of *d*_int_*_,_*_2_ = 30 μm. The period of the metamaterial array is *P* = 86 μm. The total size of the structure is 424 μm. A small-scale SRR is a scaled-down version with *d*_ext_*_,_*_1_ = 40 μm, *d*_int_*_,_*_1_ = 35 μm; *d*_ext_*_,_*_2_ = 20 μm, *d*_int_*_,_*_2_ = 15 μm. The lattice period for this dense array is *P* = 43 μm. The total size of the structure is reduced to 298 μm. This scaling of the SRR geometry is intended to shift the central frequency of the metamaterial to a higher value while maintaining the increasing absorption of the double-ring design.

The transition from a single-ring antenna to a double split-ring resonator design, while keeping the number of elements constant, resulted in a significant improvement in performance. The addition of the inner ring, which increases the total capacitance of the resonant element, leads to a slight reduction of the central frequency [20]. More importantly, it yielded a 1.5-fold increase in the total absorbed power.

**Figure 2:**
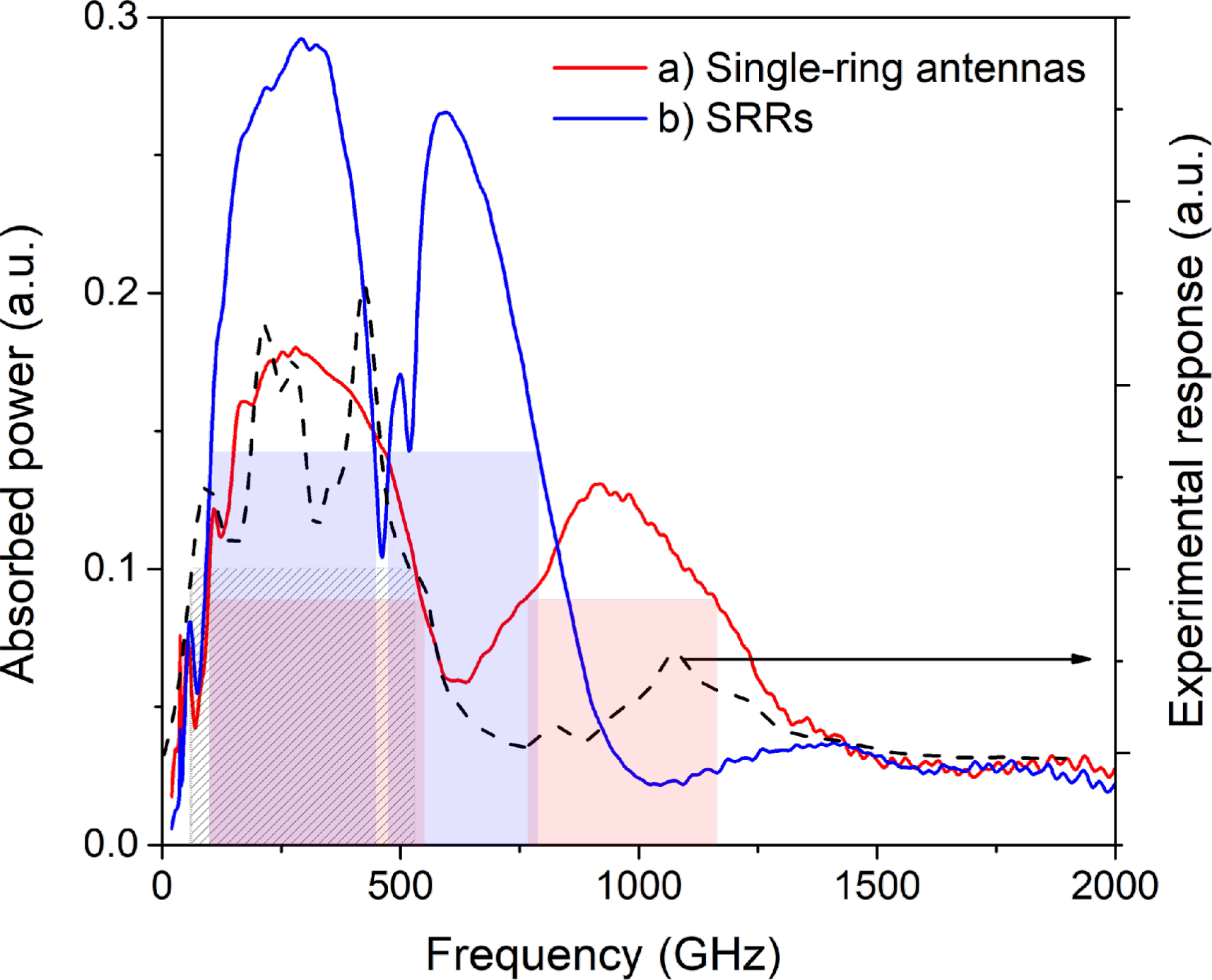
Amplitude–frequency characteristics of the metamaterial receiver. (a) 19-element array of single-ring antennas with a lattice period of *P* = 86 μm (red curve); (b) 19-element array of SRRs with *P* = 86 μm (blue curve). The dashed black curve shows the experimentally measured response of the single-ring metamaterial.

The amplitude–frequency characteristics (AFC) for the simulated single-ring and SRR designs are presented in Figure 2. For the single-ring array, the absorbed power in the first resonance maximum reached a value of 0.18 (normalized units, with 0.5 maximal total power) with the bandwidth at half maximum (FWHM) spanning from 100 to 545 GHz (Figure 2, red curve). In contrast, the SRR array demonstrated a higher absorbed power of 0.27 within a bandwidth of 105 to 440 GHz (Figure 2, blue curve). Parameters of metamaterials with CEBs and different designs are given in Table 1.

**Table 1:** Parameters of metamaterial structures with CEBs.

Design (according to Figure 1)	Type of unit cell	Number of cells	Period of structure, μm	Peak absorption, a.u.	Frequency band, GHz

a	single-ring antennas	19	86	0.18	100–545
b	split-ring resonators	19	86	0.27	105–440
c	split-ring resonators	37	43	0.25	160–820

As an experimental reference for our simulations, Figure 2 also shows the frequency response measured for a fabricated sample consisting of a 19-element single-ring metamaterial (black dashed curve). This sample had the design described in [17] and was characterized using the same experimental setup described there. This setup employs a YBaCuO Josephson junction oscillator as a broadband source, with the signal delivered to the sample via an oversized waveguide. Therefore, the measured frequency response is the combined frequency response of the entire path (oscillator, waveguide-feeder, lens and the CEB metamaterial itself), with “fingers” due to the used log-periodic antenna of the Josephson oscillator, which was not fully matched to the antenna. Despite this convolution, the experimental data clearly confirm the calculated dual-band behavior of the metamaterial, showing two broad peaks centered at approximately 350 and 1100 GHz. This agreement validates our simulation model.

**Figure 3:**
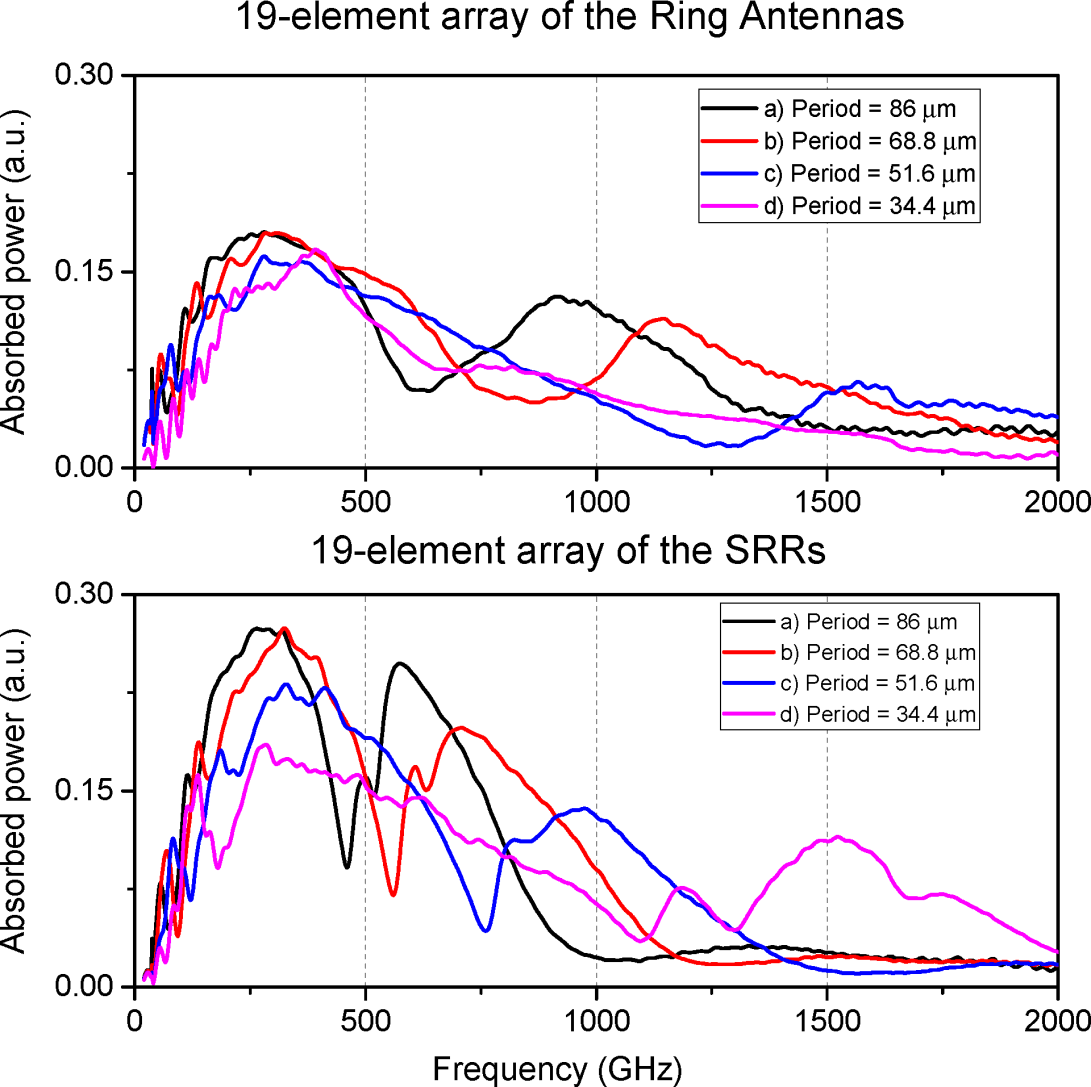
Top: AFC of the 19 single-ring antenna metamaterial for different geometric scaling factors. (a, black curve) outer ring diameter *d*_out_ = 80 μm, inner ring diameter *d*_in_ = 70 μm, period *P* = 86 μm; (b, red curve) *d*_out_ = 64 μm, *d*_in_ = 56 μm, *P* = 68.8 μm; (c, blue curve) *d*_out_ = 48 μm, *d*_in_ = 42 μm, *P* = 51.6 μm; (d, purple curve) *d*_out_ = 32 μm, *d*_in_ = 28 μm, *P* = 34.4 μm. Bottom: AFC of the 19 SRR-based metamaterial for different geometric scaling factors. The design parameters and scaling factors (0%, 20%, 40%, and 60%) correspond to the upper plot.

The AFC of the single-ring and SRR metamaterials with various scaling factors are presented in Figure 3. The optimal number and size of the resonators are governed by the requirement to fill the Airy spot of the silicon lens. If the total array size is smaller than the Airy spot, a portion of the incident signal will not interact with the metamaterial, instead scattering into the surrounding space. Our simulations confirm this principle: A reduction in the SRR dimensions and the array period by 20% led to a broadening of the absorption bandwidth and a small shift of the first resonance maximum towards higher frequencies. A further reduction of dimensions by 40% resulted in an even wider bandwidth; however, the peak absorbed power began decreasing, indicating that the array size was becoming insufficient relative to the Airy spot. A drastic 60% size reduction caused a severe deterioration of absorption.

**Figure 4:**
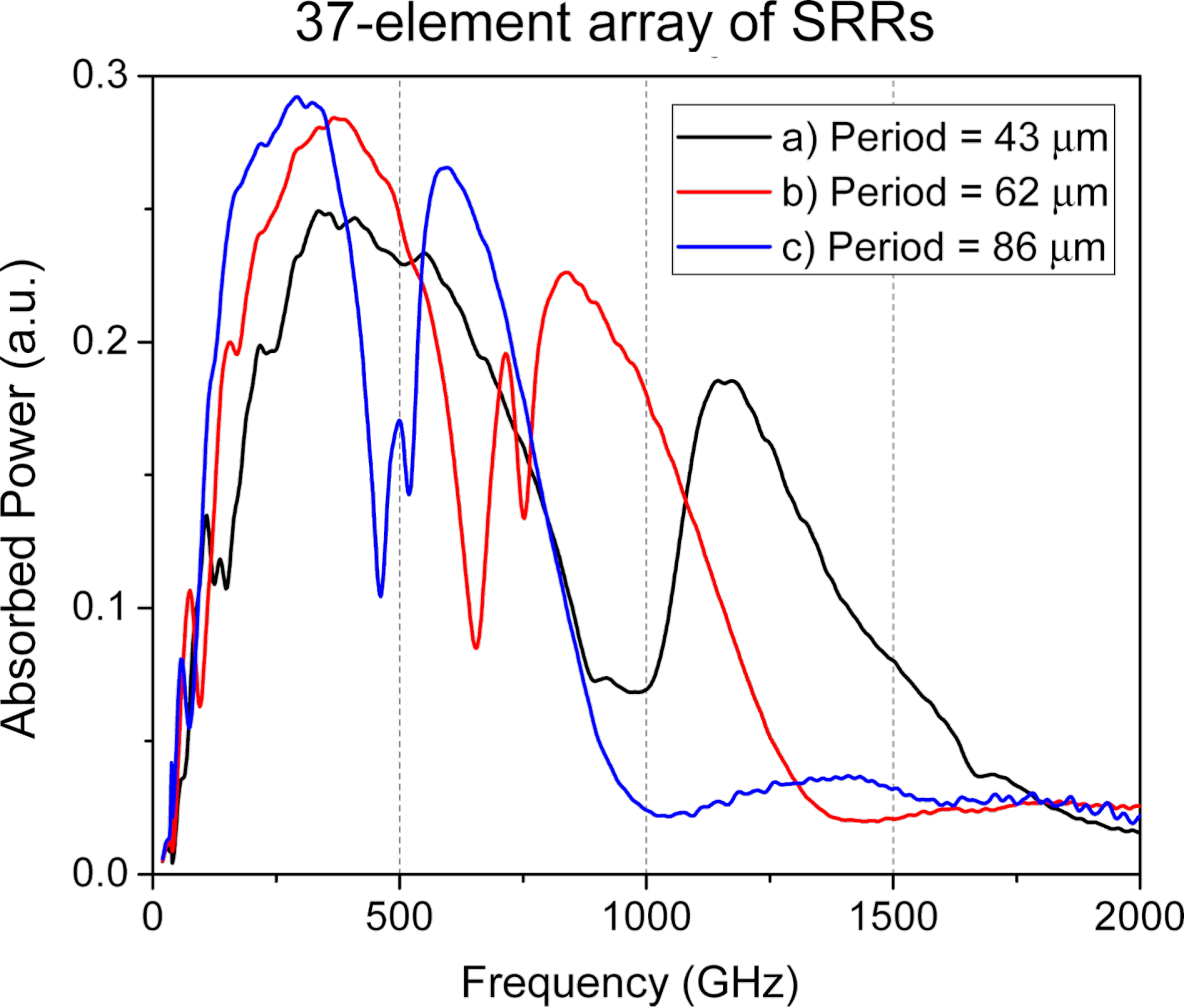
The amplitude–frequency characteristics of the 37-element array of SRR-based metamaterial for different periods of the lattice.

To achieve the widest possible bandwidth using SRRs, our results shown in Figure 3 suggest prioritizing somewhat smaller unit cell sizes. Simply scaling down a fixed 19-element array leads to less efficient signal reception since the array is becoming smaller than the Airy spot. As an efficient alternative, we propose to halve the SRR dimensions and array period while simultaneously increasing the number of elements from 19 to 37 (Figure 1c). This approach successfully increased the absorbed power to 0.25, which is by a factor of 1.4 higher than that of the single-ring array, while also achieving an ultrawide receiving band from 160 to 820 GHz (Figure 4, black line). If the 37-element array structure occupies the same area as the original single-ring structure, larger absorption efficiency at the first peak can be achieved (Figure 4, red line), but the working bandwidth will be narrower than for the structure with smaller rings. Thus, by selecting the overall structure size, a compromise can be found between the maximum absorption efficiency and the widest receiving bandwidth.

**Figure 5:**
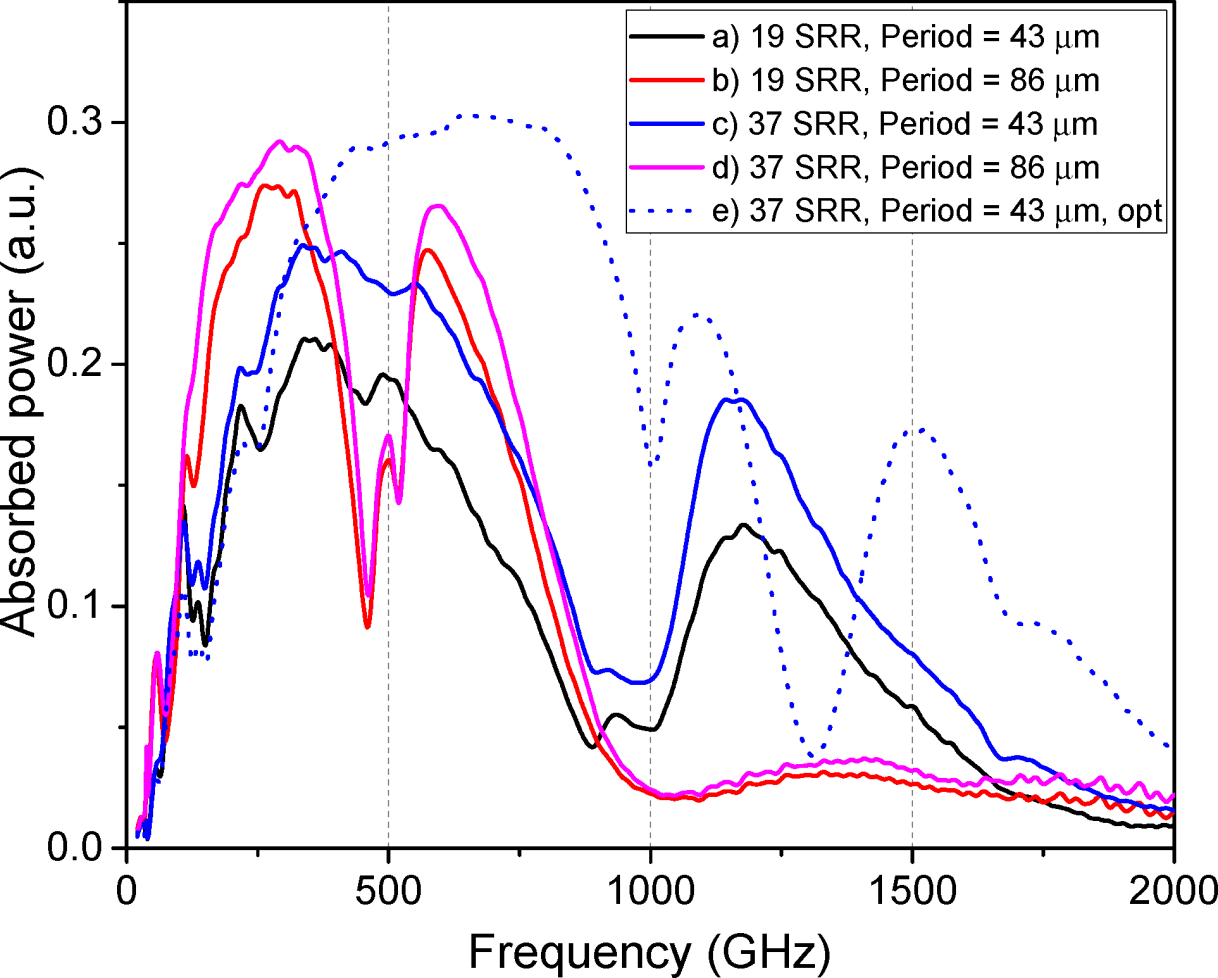
Dependence of the absorbed power on the number of elements in the SRR array.

It is important to note that the choice of the number of receiving antennas should be in a proper balance. Although a larger array can better fill the Airy spot, it also increases the total number of bolometers. This, in turn, increases the differential resistance of the structure at the operating point and increases the current noise contribution of the readout amplifier [17,22]. Furthermore, a larger number of elements increases the fabrication complexity. Crucially, nearly doubling the number of elements (from 19 to 37) does not produce a proportional increase in the absorbed power (Figure 5).

Figure 5 shows the AFC of the SRR metamaterial with a different number of elements. For the large-scale design (period *P* = 86 μm, rings: *d*_out_*_,_*_1_/*d*_in_*_,_*_1_ = 80/70 μm, *d*_out_*_,_*_2_/*d*_in_*_,_*_2_ = 40/30 μm), doubling the number of elements increases the absorbed power by about 7% only, with a minor increase in bandwidth. The same doubling for the miniaturized design (*P* = 43 μm, rings: *d*_out_*_,_*_1_/*d*_in_*_,_*_1_ = 40/35 μm, *d*_out_*_,_*_2_/*d*_in_*_,_*_2_ = 20/15 μm) is more efficient, leading to 17% increase in power. This higher efficiency is directly linked to the Airy spot coverage: Adding elements to the smaller array more effectively increases its total area towards the optimal size. For the already large array, new elements are added at the periphery or outside the most intense part of the Airy spot, which does not actually help.

The obtained results can be further enhanced through optimized design parameters of the structure. Specifically, increasing the substrate thickness to 700 μm enables an increase in absorbed power up to 0.3 a.u. across an ultrabroad frequency range of 200–1200 GHz (Figure 5e). Such a thick substrate can be realized by using commercially available substrates with greater thickness (or by stacking and bonding multiple thinner substrates), or by employing a lens with a pedestal structure.

## Discussion

Solving the problem of broadband high-sensitivity reception for terahertz applications naturally entails comparing the metamaterial-based approach presented here with traditional broadband antenna solutions such as the log-periodic [23–25] or spiral antennas [26–27]. These antennas are indeed a well-established technology, providing wideband frequency response and high detection/radiation efficiency. However, their widespread use is subject to a fundamental limitation: The active receiving element is typically a single detector unit located at the antenna’s feed point. This configuration can become a bottleneck when detecting ultralow power signals in the presence of high background radiation, as the single detector must handle the entire power load, potentially limiting the dynamic range and complicating the optimization of noise-equivalent power (NEP).

There have been proposals to integrate multiple sensing elements directly into the structure of a log-periodic antenna [28–30]. While promising, such designs face significant challenges in implementation. The complex geometry of the antenna makes it difficult to integrate a large number of detectors and to design complex series–parallel electrical networks necessary for optimal power distribution and impedance matching. In contrast, the metamaterial approach offers a fundamentally more flexible paradigm. A periodic array of resonators, such as our SRR-based design, inherently functions as a multiabsorber system. This architecture allows for the precise engineering of the detector network, that is, the number of CEBs, their individual connection (series or parallel), and the overall array configuration to achieve an optimal balance between power load, responsivity, and total noise [17,22].

This capability is particularly critical for applications like cosmic microwave background polarimetry or high-resolution spectroscopy, where the detector must operate photon-noise-limited under a specific background power load. For CEBs, we have previously demonstrated that the optimal configuration for minimizing the total NEP with a given readout amplifier involves a specific series–parallel combination of bolometers. The metamaterial platform is ideal for implementing such an optimized multiabsorber receiver. By adapting the array geometry and the electrical connection scheme between CEBs, one can precisely control the power absorbed per bolometer and the resulting differential resistance, thereby achieving photon-noise-limited performance across a wide bandwidth. This level of design control is considerably more challenging to realize within the constrained geometry of a single-feed log-periodic antenna.

## Conclusion

In this work, we have presented a comprehensive electromagnetic study on the design and optimization of a metamaterial receiver based on split-ring resonators integrated with cold-electron bolometers. The transition from a conventional single-ring antenna design to a double SRR configuration has been demonstrated to be a highly efficient strategy to enhance the receiver performance. This design improvement resulted in a substantial 1.5-fold increase in the absorbed power, confirming the theoretical advantage of SRRs in providing a stronger magnetic resonance and greater field concentration within the capacitive gaps where the CEBs are located.

Our investigation of the scaling of the metamaterial array revealed a critical design trade-off. While reducing the dimensions of the SRR unit cells effectively broadens the operational bandwidth, it also reduces the total absorbed power if the array’s physical size becomes smaller than the Airy spot of the coupling lens. We successfully resolved this issue by implementing a strategy of simultaneous miniaturization and increasing the array density. By halving the SRR dimensions and lattice period while nearly doubling the number of elements (from 19 to 37), we achieved an optimal compromise. The resulting receiver exhibits both enhanced absorption (by a factor of 1.4 larger than the original single-ring design) and an ultrawide bandwidth spanning from 160 to 820 GHz.

Furthermore, we quantified the non-linear relationship between the number of array elements and the absorbed power, showing that the benefit of adding elements is significantly higher for a miniaturized array that initially underfills the Airy spot. This provides a crucial practical guideline for designing efficient multiabsorber receivers, balancing performance gains against the increased technological complexity and noise considerations associated with a larger number of bolometers.

This work solidifies the position of CEB-based SRR metamaterials as a highly promising platform for constructing ultrabroadband, high-sensitivity receivers essential for next-generation spectroscopic and radioastronomical applications, particularly in demanding space and balloon-borne environments. One more important potential application for such broadband receiving system is the use for axion search experiments with broadband coaxial dish antennas [31–32]. Future work will focus on the experimental fabrication and characterization of the proposed miniaturized 37-element SRR array to validate these simulation results.

## Data Availability

Data generated and analyzed during this study is available from the corresponding author upon reasonable request.

## References

[1] Ajito K, Nakamura M, Tajima T, Ueno Y, Lindon J C, Tranter G E, Koppenaal D W (2017). Terahertz Spectroscopy Methods and Instrumentation. Encyclopedia of Spectroscopy and Spectrometry.

[2] Likhachev S F, Larchenkova T I (2024). Phys-Usp.

[3] Maffei B, Aghanim N, Aumont J, Battistelli E, Beelen A, Besnard A, Borgo B, Calvo M, Catalano A, Chluba J, Zmuidzinas J, Gao J-R (2024). BISOU: a balloon pathfinder for CMB spectral distortions studies. Millimeter, Submillimeter, and Far-Infrared Detectors and Instrumentation for Astronomy XII.

[4] Coulon X, Maffei B, Aghanim N (2024). EPJ Web Conf.

[5] Novikov D I, Doroshkevich A G, Larchenkova T I, Malinovsky A M, Mihalchenko A O, Osipova A M, Parfenov K O, Pilipenko S V (2025). Phys-Usp.

[6] Anghel D V, Kuzmin L S (2020). Phys Rev Appl.

[7] Pimanov D A, Pankratov A L, Gordeeva A V, Chiginev A V, Blagodatkin A V, Revin L S, Razov S A, Safonova V Yu, Fedotov I A, Skorokhodov E V (2025). Supercond Sci Technol.

[8] Nahum M, Martinis J M (1995). Appl Phys Lett.

[9] Irwin K D, Hilton G C (2005). Transition-Edge Sensors. Cryogenic Particle Detection.

[10] Withington S (2022). Contemp Phys.

[11] Safonova V Y, Gordeeva A V, Blagodatkin A V, Pimanov D A, Yablokov A A, Pankratov A L (2024). Beilstein J Nanotechnol.

[12] O'Brient R, Ade P, Arnold K, Edwards J, Engargiola G, Holzapfel W L, Lee A T, Myers M J, Quealy E, Rebeiz G (2013). Appl Phys Lett.

[13] Gordeeva A V, Pankratov A L, Pugach N G, Vasenko A S, Zbrozhek V O, Blagodatkin A V, Pimanov D A, Kuzmin L S (2020). Sci Rep.

[14] Pimanov D A, Frost V A, Blagodatkin A V, Gordeeva A V, Pankratov A L, Kuzmin L S (2022). Beilstein J Nanotechnol.

[15] Lemziakov S A, Karimi B, Nakamura S, Lvov D S, Upadhyay R, Satrya C D, Chen Z-Y, Subero D, Chang Y-C, Wang L B (2024). J Low Temp Phys.

[16] Salatino M, de Bernardis P, Kuzmin L S, Mahashabde S, Masi S (2014). J Low Temp Phys.

[17] Revin L S, Pimanov D A, Pankratov A L, Blagodatkin A V, Matrozova E A, Chiginev A V, Gordeeva A V, Fedotov I A, Skorokhodov E V, Gusev N S (2024). Phys Rev Appl.

[18] Sydoruk O, Tatartschuk E, Shamonina E, Solymar L (2009). J Appl Phys.

[19] Pendry J B, Holden A J, Robbins D J, Stewart W J (1999). IEEE Trans Microwave Theory Tech.

[20] Reddy A N, Raghavan S (2013). Split ring resonator and its evolved structures over the past decade: This paper discusses the nuances of the most celebrated composite particle (split-ring resonator) with which novel artificial structured materials (called metamaterials) are built. 2013 IEEE International Conference ON Emerging Trends in Computing, Communication and Nanotechnology (ICECCN).

[21] Marqués R, Martín F, Sorolla M (2008). Metamaterials with Negative Parameters: Theory, Design and Microwave Applications.

[22] Kuzmin L S, Pankratov A L, Gordeeva A V, Zbrozhek V O, Shamporov V A, Revin L S, Blagodatkin A V, Masi S, de Bernardis P (2019). Commun Phys.

[23] Tarasov M, Kuzmin L, Stepantsov E, Kidiyarova-Shevchenko A (2006). Quasioptical Terahertz Spectrometer Based on a Josephson Oscillator and a Cold Electron Nanobolometer. Nanoscale Devices - Fundamentals and Applications.

[24] Stepantsov E, Tarasov M, Kalabukhov A, Kuzmin L, Claeson T (2004). J Appl Phys.

[25] Gao X, Zhang T, Du J, Weily A R, Guo Y J, Foley C P (2017). Supercond Sci Technol.

[26] Tretyakov I V, Khudchenko A V, Rudakov K I, Ivashentseva I V, Kaurova N S, Voronov B M, Kirsanova M S, Larchenkova T I, Goltsman G N, Baryshev A M (2025). IEEE Trans Terahertz Sci Technol.

[27] Malnou M, Luo A, Wolf T, Wang Y, Feuillet-Palma C, Ulysse C, Faini G, Febvre P, Sirena M, Lesueur J (2012). Appl Phys Lett.

[28] Yu M, Geng H, Hua T, An D, Xu W, Chen Z N, Chen J, Wang H, Wu P (2020). Supercond Sci Technol.

[29] Sharafiev A, Malnou M, Feuillet-Palma C, Ulysse C, Wolf T, Couëdo F, Febvre P, Lesueur J, Bergeal N (2018). Supercond Sci Technol.

[30] Glushkov E I, Chiginev A V, Kuzmin L S, Revin L S (2022). Beilstein J Nanotechnol.

[31] Knirck S, Hoshino G, Awida M H, Cancelo G I, Di Federico M, Knepper B, Lapuente A, Littmann M, Miller D W, Mitchell D V (2024). Phys Rev Lett.

[32] Hoshino G, Knirck S, Awida M H, Cancelo G I, Corrodi S, Di Federico M, Knepper B, Lapuente A, Littmann M, Miller D W (2025). Phys Rev Lett.

